# Metagenome-assembled Genomes of Six Novel Ammonia-oxidizing Archaea (AOA) from Agricultural Upland Soil

**DOI:** 10.1264/jsme2.ME22035

**Published:** 2022-08-11

**Authors:** Huicheng Zhao, Linqi Zhang

**Affiliations:** 1 Key Laboratory of Agricultural Water Resources, Center for Agricultural Resources Research, Institute of Genetics and Developmental Biology, Chinese Academy of Sciences, Shijiazhuang, 050021, China; 2 University of Chinese Academy of Sciences, Beijing 100039, China

**Keywords:** metagenome-assembled genome, AOA, Thaumarchaeota, agricultural upland soil

## Abstract

Ammonia-oxidizing archaea (AOA), key players in agricultural upland soil nitrification, convert soil ammonium to nitrite. The microbial oxidation of ammonia to nitrite is an important part of the global biogeochemical nitrogen cycle. In the present study, we recovered six novel AOA metagenome-assembled genomes (MAGs) containing genes for carbon (C) fixation and nitrogen (N) metabolism by using a deep shotgun metagenomic sequencing strategy. We also found that these AOA MAGs possessed cobalamin synthesis genes, suggesting that AOA are vitamin suppliers in agricultural upland soil. Collectively, the present results deepen our understanding of the metabolic potential and phylogeny of AOA in agroecosystems.

The microbial oxidation of ammonia is the first and rate-limiting step of nitrification, which is central to the biogeochemistry of the soil nitrogen cycle (Kuypers *et al.*, 2018). In nature, the oxidation of ammonia (NH_3_) to nitrite (NO_2_^–^) is catalyzed by ammonia-oxidizing bacteria (AOB), ammonia-oxidizing archaea (AOA), and complete ammonia oxidizers (comammox) (Martens-Habbena *et al.*, 2009; Daims *et al.*, 2015). Ammonia oxidizers control the rate of ammonia oxidation by regulating substrate supply and adaptation to growth under specific conditions, and the discovery of AOA has expanded our understanding of the nitrogen cycle. AOA are widely distributed in the natural environment (Könneke *et al.*, 2005; Leininger *et al.*, 2006; Hatzenpichler *et al.*, 2008) and play an important role in global nitrogen and carbon cycling (Wuchter *et al.*, 2006). A previous study demonstrated that the archaeal *amoA* gene diversified into five major lineages, i.e., *Nitrosopumilus*, *Nitrosotalea*, *Nitrosocaldus*, *Nitrososphaera*, and ‘*Nitrososphaera* sister’ (Pester *et al.*, 2012), and all clusters belonged to the phylum *Thaumarchaeota*.

The ecological niches of AOB and AOA have also been shown to depend on the soil ammonia concentration, pH, and other environmental factors (Ando *et al.*, 2009; Onodera *et al.*, 2010; Morimoto *et al.*, 2011). AOA generally prefer low concentrations of ammonia and acidic environments (Zhang *et al.*, 2012), whereas AOB exhibit a preference for environments with high concentrations of ammonia (Verhamme *et al.*, 2011). However, *Nitrososphaera viennensis* isolated from garden soil was found to have higher NH_3_ tolerance than the marine isolate *Nitrosopumilus maritimus* (Tourna *et al.*, 2011), and its optimum growth pH was 7.5. A neutrophilic AOA of Thaumarchaeal Group I.1a may also oxidize ammonia in the presence of up to 5‍ ‍mM ammonia (Jung *et al.*, 2014). A novel ureolytic AOA isolated from a pH 7.5 arable soil, named *Candidatus* Nitrosocosmicus franklandus, which belongs to the ‘*Nitrososphaera* sister’ group, was recently shown to survive under high ammonia concentrations (Lehtovirta-Morley *et al.*, 2016). These findings indicate that AOA inhabit diverse environments, and the ammonia oxidation activity of AOA may not be limited by soil pH or the concentration of ammonia.

The majority of studies on AOA have mainly involved taxonomic-based approaches, such as 16S rRNA gene metabarcoding or real-time PCR. However, these approaches only focus on the capture of taxonomic information and community abundance. In the present study, we collected agricultural upland soils from the surface layer (0–20‍ ‍cm) in Herbin, Shenyang, Quzhou, Shangqiu, and Changwu in northern China. Soil total DNA was extracted from 0.5‍ ‍g soil using an Omega Mag-Bind Soil DNA Kit (Omega Biotek) following the user’s manual. DNA libraries were prepared using the KAPA HyperPlus Kit (Roche Sequencing). A deep shotgun metagenomic sequencing strategy (an average of 47 Gb for each sample) was used to recover metagenome-assembled genomes (MAGs) by using MetaBAT2 (v2.10.2) runMetaBat.sh parameters (Kang *et al.*, 2019) and CONCOCT (v1.1.0) (Alneberg *et al.*, 2014) binning tools to infer the metabolic potential as well as phylogeny of AOA. We obtained 563 non-redundant MAGs using dRep dereplicate parameters (Olm *et al.*, 2017). Clean reads were individually assembled into contigs using MEGAHIT (v1.0.2) with meta-large parameters (Li *et al.*, 2015). The completeness and contamination of MAGs were estimated by the CheckM (v1.1.3) lineage_wf function (Parks *et al.*, 2015). Prokka was used for gene prediction and annotation (Seemann, 2014). A maximum likelihood tree using the LGGAMMA model was generated using FastTree (v.2.1.10; parameters: -lg -gamma) (Price *et al.*, 2010). Abundance is expressed as “genome copies per million reads” and was calculated using metawrap quant_bin parameters (Uritskiy *et al.*, 2018).

According to genome taxonomic and functional annotation results, six MAGs (ThauHEB1, ThauHEB2, ThauQZ2, ThauCW1, ThauSQ3, and ThauSY3) recovered from assembly data were assigned to AOA groups. ThauHEB1 and ThauHEB2 were obtained from Harbin, ThauQZ2 from Quzhou, ThauCW1 from Changwu, ThauSQ3 from Shangqiu, and ThauSY3 from Shenyang. The completeness of MAGs ranged from between 52.05 and 96.76% and contamination from between 0.97 and 8.58%. The completeness of ThauQZ2 and ThauHEB2 were 96.76 and 93.05%, respectively, while the contamination of these two MAGs were 1.94 and 2.51%, respectively, which suggested that ThauQZ2 and ThauHEB2 represent nearly complete AOA genomes. The genome sizes of our six MAGs varied from 0.985 to 2.573‍ ‍Mb and the GC contents varied from 28.75 to 48.85%. The GC content of the nucleic acid sequence is a fundamental mole­cular trait that may be used as an indicator to reflect the evolutionary characteristics of AOA, possibly representing different adaptation traits for responses to environmental heterogeneity. The estimated numbers of CDSs (coding sequences) were 1,499 for ThauHEB1, 3,170 for ThauHEB2, 3,119 for ThauQZ2, 2,237 for ThauCW1, 1,447 for ThauSQ3, and 1,899 for ThauSY3. The percentages of CDSs with functions, *i.e.*, approximately 58–81%, were predicted using the eggNOG database (Huerta-Cepas *et al.*, 2019) of these genes, and 28–53% were predicted using the KEGG database (Kanehisa and Goto, 2000). The number of total RNA genes varied from 44 to 76 in all six MAGs. Basic information on these AOA MAGs is listed in Supplementary Table S1.

Our six AOA MAGs were in different archaeal clades (Fig. 1). For example, ThauHEB1 and ThauQZ2 were placed in the same clade of the phylogenetic tree, while ThauCW1, ThauHEB2, ThauSY3, and ThauSQ3 were placed in another major clade of the phylogenetic tree. ThauHEB1 from chernozem soil (pH=5.9) had a close genetic relationship with the clade of *Nitrosotalea*, and species belonging to the *Nitrosotalea* lineage were generally associated with nitrification activity in acidic environments (Herbold *et al.*, 2017). ThauCW1 from dark loessial soil (pH=8.0) was placed in the clade of the genus *Nitrososphaera*, which mostly inhabit alkaline environments (Lehtovirta-Morley *et al.*, 2016), and ThauCW1 was the most abundant MAG among other AOA MAGs (Supplementary Table S1). ThauHEB2, ThauSY3, and ThauSQ3 were placed in the same clade as the order *Nitrososphaerales*. ThauQZ2 had a close genetic relationship with the Nitrososphaerales archaeon TH5894, and these MAGs were considered to be novel clades in AOA populations. The closest placement average nucleotide identity (ANI) with the reference species of four MAGs (ThauHEB1, ThauCW1, ThauSQ3, and ThauQZ2) varied from between 77.05 and 92.47%, and ThauHEB2 and ThauSY3 MAGs had no species that were assigned in the GTDB database (R95) (Parks *et al.*, 2020).

CO_2_ fixation is the most important biosynthesis process for autotrophs to synthesize their entire biomass from inorganic carbon. We found that our AOA MAGs had genes related to the hydroxypropionate/hydroxybutyrate (HP/HB) cycle (Fig. 2a), which were responsible for the assimilation of CO_2_ and HCO_3_^–^ in the environment. The HP/HB cycle is present in the majority of reported Thaumarchaeal genomes (Walker *et al.*, 2010; Tourna *et al.*, 2011; Jung *et al.*, 2014; Könneke *et al.*, 2014; Kerou *et al.*, 2016), and this pathway has higher energy efficiency than other aerobic autotrophic pathways for fixing inorganic carbon in aerobic environments (Könneke *et al.*, 2014). The genes encoding mcr, K1503B, and K14465 proteins were not found in AOA MAGs, and we speculated that these MAGs may contain unknown genes participating in the HP/HB pathway. The previously reported growth rates of an ammonia-oxidizing archaeon from soil were strongly enhanced when pyruvate was added (Tourna *et al.*, 2011). Furthermore, we found that the *pycA* gene catalyzed pyruvate carboxylase in all six AOA MAGs (Fig. 2a), indicating that our AOA MAGs from soil environments exhibited similar pyruvate metabolic potential to other AOA lineages.

We also examined associated genes responsible for the N cycle process, such as ammonia oxidation and ammonia assimilation, in our AOA MAGs (Fig. 2b). We found that ThauHEB1 had *amoA*, *amoB*, and *amoC* genes; ThauQZ2 and ThauHEB2 had *amoA* and *amoB* genes; and ThauSQ3, ThauSY3, and ThauCW1 had *amoA* gene. In addition, ThauHEB1, ThauHEB2, ThauQZ2, and ThauCW1 possessed the *nirK* gene. Although previous studies reported structural variations between archaeal and bacterial copper-containing nitrite reductase (NirK) enzymatic properties (Kobayashi *et al.*, 2018), AOA and some bacteria had *nirK gene* and are phylogenetically and functionally relevant. Genes including the *ureC* gene encoding urease were also detected in all six MAGs, and urease was shown to catalyze the hydrolysis of urea to ammonium and CO_2_ (Oshiki *et al.*, 2018). We found the *glnA* gene in ThauHEB1, ThauHEB2, ThauQZ2, ThauCW1, and ThauSY3, which is the key gene for ammonia assimilation to L-glutamine and L-glutamate in glutamate metabolism, and this pathway is regarded as the main transport and storage form of ammonia. ThauHEB1, ThauHEB2, ThauQZ2, and ThauCW1 also had the *gdhA* gene encoding L-glutamate dehydrogenase to catalyze the oxidative deamination of glutamate to 2-oxoglutarate, which may also provide the substrate for ammonia oxidation.

Previous studies reported that Thaumarchaeota play an important role in cobalamin biosynthesis (vitamin B12) in aquatic environments (Doxey *et al.*, 2015). The involvement of AOA from agricultural upland soils in cobalamin production remains unclear. We identified the associated genes in the cobalamin synthesis pathway and found that our AOA MAGs were predominantly from the anaerobic pathway and had a widespread genetic capacity for the synthesis of cobalamin. For example, the gene *cbiA* involved in cobyrinic acid transformation to cobyrinic acid a,c-diamide cobyrinate was found in ThauHEB2, ThauSY3, ThauQZ2, and ThauCW1; the gene *cobA* involved in cobyrinic acid a,c-diamide transformation to adenosyl cobyrinate a,c-diamide was identified in all six AOA MAGs; genes (*cobQ*; *cobC*/*cobD*; *cobY*; *cobS*) involved in adenosyl cobyrinate a,c-diamide transformation to adenosyl cobyrinate hexaamide, adenosylcobinamide, adenosine-GDP-cobinamide, and Vitamin B_12_ were detected in ThauHEB1, ThauHEB2, ThauSY3, ThauQZ2, and ThauCW1; and other genes (*cbiC/cbiD/cbiE/cbiF/cbiG/cbiH/cbiL/cbiT*) involved in intermediate transformation, such as Factor, Co-Factor, and Co-Precorrin, were also found in these AOA MAGs. In addition, all six AOA MAGs had genes involved in assimilatory sulfate reduction, which suggests that the activity of AOA is accompanied by sulfur metabolism. Sulfur assimilation to synthesize cysteine molecules is apparently essential for all forms of life, including archaea, bacteria, and eukaryotes. Specifically, ThauHEB1 and ThauQZ2 had the full set of genes responsible for the sulfate reduction pathway, successively converting sulfate to adenosine 5′-phosphosulfate (APS), 3′-phosphoadenosine 5′-phosphosulfate (PAPS), sulfite, and sulfide, including *sat* encoding sulfate adenylyltransferase, *cysC* encoding adenylylsulfate kinase or related kinase sulfates, *cysH* encoding phosphoadenosine phosphosulfate reductase, and *sir* encoding sulfite reductase. However, the remaining MAGs had a truncated sulfate reduction pathway; for example, ThauHEB2 had *sat*, *cysH*, and *sir*; ThauSY3 had *sat*, *cysC*, and *cysH*; ThauSQ2 had *sat* and *sir*; and ThauCW1 had *cysC*, *cysH*, and *sir*.

## Data Availability Statement

Raw data are available at the National Center for Biotechnology Information (NCBI) under BioProject PRJNA792913, with the accession numbers SRR17477666, SRR17477667, SRR17477668, SRR17477669, and SRR17477670. The accession numbers of the metagenome assembled genomes are JAKEIF000000000, JAKEIG000000000, JAKEIH000000000, JAKEII000000000, JAKEIJ000000000, and JAKEIK000000000.

## Citation

Zhao, H., and Zhang, L. (2022) Metagenome-assembled Genomes of Six Novel Ammonia-oxidizing Archaea (AOA) from Agricultural Upland Soil. *Microbes Environ ***37**: ME22035.

https://doi.org/10.1264/jsme2.ME22035

## Supplementary Material

Supplementary Material

## Figures and Tables

**Fig. 1.**
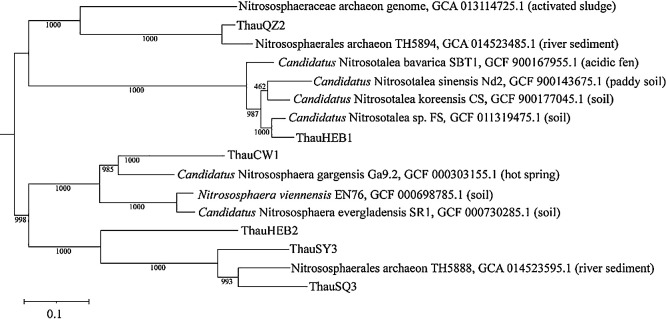
Phylogeny of ThauHEB1, ThauHEB2, ThauQZ2, ThauCW1, ThauSQ3, and ThauSY3 with 43 concatenated marker proteins of archaea. The tree was constructed using the maximum-likelihood method. The scale bar indicates genetic distance. Numbers at the nodes are bootstrap values (1,000 replicates).

**Fig. 2.**
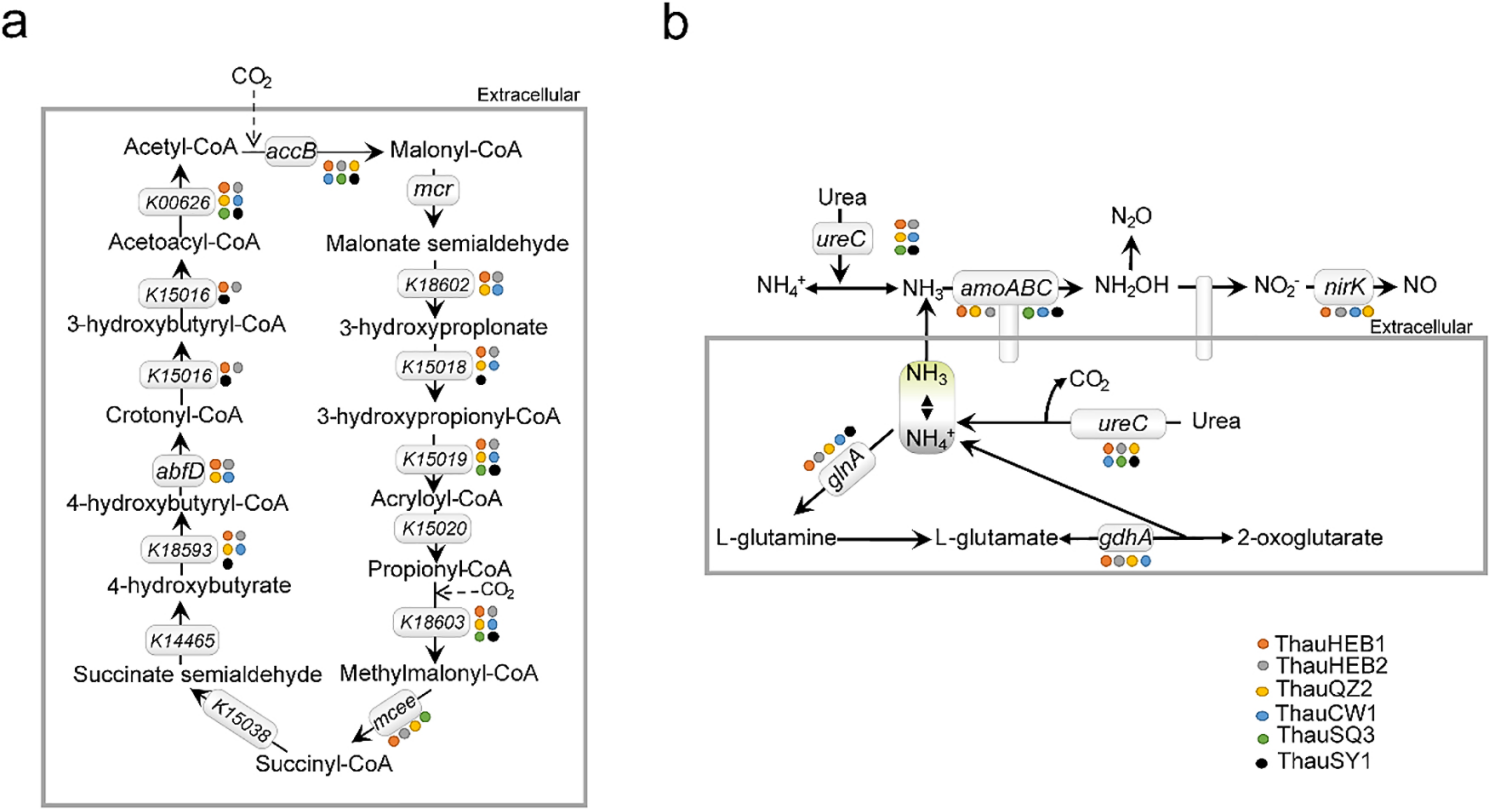
The ecological functions of AOA include carbon fixation and nitrogen metabolism. (a) Carbon fixation by hydroxypropionate/hydroxybutyrate (HP/HB) cycle and (b) ammonium oxidation and assimilatory. MAGs (denoted by a circle) harboring corresponding genes are marked in the figure, and the circle color represents different MAGs, *i.e.*, ThauHEB1, ThauQZ2, ThauQZ2, ThauCW1, ThauSQ3, and ThauSY3.
