# Supplementary material for: Metagenome-assembled Genomes of Six Novel Ammonia-oxidizing Archaea (AOA) from Agricultural Upland Soil
Source: Microbes Environ. 2022 Aug 11;37(3):ME22035. doi: 10.1264/jsme2.ME22035 (PMC9530722; doi:10.1264/jsme2.ME22035)
Supplement: Supplementary file 1 — Supplementary Material [file 37_22035_s1.pdf]

Supplementary Table 1. Comparison of the general genome features of six AOA MAGs.

	**ThauHEB1**	**ThauHEB2**	**ThauQZ2**	**ThauCW1**	**ThauSQ3**	**ThauSY3**
Genome size, bp	1,135,571	2,565,190	2,573,798	1,705,448	985,225	1,377,005
Longest contig, kb	30.590	52.615	25.606	28.558	13.163	50.990
Completeness, %	84.07	93.05	96.76	80.03	52.05	71.28
Contamination, %	0.97	2.51	1.94	2.75	8.58	6.31
G + C content, %	37.86	37.75	28.75	48.85	35.06	34.88
Total CDSs, n	1499	3170	3119	2237	1447	1899
Predicted with the eggNOG database, n (%)	1221 (81.45)	1854 (58.48)	1854 (59.44)	1534 (68.57)	974 (67.31)	1295 (68.195)
Predicted with the KEGG database, n (%)	795 (53.04)	534 (28.80)	777 (41.91)	643 (41.92)	355 (36.48)	519 (40.08)
Total RNA genes, n	65	76	74	68	44	61
MAG abundance (genome copies per million reads)	0.076	0.136	0.134	0.147	0.143	0.142
